# Supplementary material for: The Evolution and Characterization of the RNA Interference Pathways in Lophotrochozoa
Source: Genome Biol Evol. 2024 May 7;16(5):evae098. doi: 10.1093/gbe/evae098 (PMC11114477; doi:10.1093/gbe/evae098)
Supplement: evae098_Supplementary_Data [file evae098_supplementary_data.zip › Supplementary_materials.zip!/selected_miRNAs/NoGe_selected_miRNAs/mirdeep_pdf_files/NMRB01000177.1_Notospermus_geniculatus_strain_Ushimado_scaffold177,_whole_genome_shotgun_sequence_6426.pdf]

Provisional ID	:	NMRB01000177.1_Notospermus_geniculatus_strain_Ushimado_scaffold177,_whole_genome_shotgun_sequence_6426
Score total	:	1320.1
Score for star read(s)	:	3.9
Score for read counts	:	1312.4
Score for mfe	:	0.8
Score for randfold	:	0
Score for cons. seed	:	3
Total read count	:	2586
Mature read count	:	2492
Loop read count	:	0
Star read count	:	94

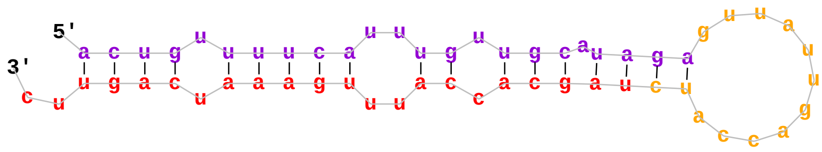

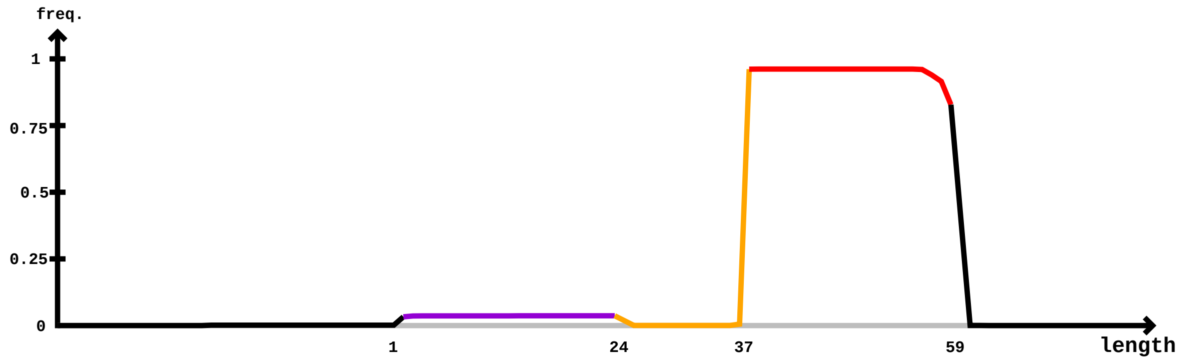


```
                                     Star                                 Mature
5'- ggagacuuuuuggccgugauugccugucaaguguacuguuuucauuuguugcauagaguuauugaccaucuagcaccauuugaaaucaguucauuggagaggacaugcugcg   -3'    obs
    ggagacuuuuuggccgugauugccugucaaguguacuguuuucauuuguugcauagaguuauugaccaucuagcaccauuugaaaucaguucauuggagaggacaugcugcg          exp
    ............((.((...(((((.((.((((.((((.(((((..((.(((.((((...........))))))).))..))))).)))).)))).)).))).)).)).)).    reads   mm   sample
    ..................................acuguuuucauuuguugcauaga.......................................................    29      0    Fm2
    ..................................acuguuuucauuAguugcauaga.......................................................    1       1    Fm2
    ...................................cuguuuucauuuguugcauaga.......................................................    6       0    Fm2
    .....................................................................cuGgcaccauuugaaaucaguu.....................    12      1    Fm2
    ......................................................................uagcaccauuugaaaucUguu.....................    1       1    Fm2
    ......................................................................uagcaccauuugaaaucaguu.....................    7       0    Fm2
    ......................................................................uagcaccauuugaaaucCguuc....................    1       1    Fm2
    ......................................................................uagcaccauuugaaaucaguuc....................    328     0    Fm2
    ......................................................................uagcaccauuugaaauAaguuc....................    2       1    Fm2
    ......................................................................uagcaccauuugaaaucUguuc....................    1       1    Fm2
    ......................................................................uagcGccauuugaaaucaguuc....................    1       1    Fm2
    ......................................................................uUgcaccauuugaaaucaguuc....................    1       1    Fm2
    ......................................................................uagcaccauuAgaaaucaguuc....................    1       1    Fm2
    ......................................................................uagcaccauuugaaaucaguGca...................    10      1    Fm2
    ......................................................................uagcaccauuugaaaucaguucU...................    9       1    Fm2

    ..................................acuguuuucauuuguugcauaga.......................................................    10      0    Fm1
    ......................................................................uagcaccauuugaaauca........................    4       0    Fm1
    ......................................................................uagcaccauuugaaaucaguu.....................    3       0    Fm1
    ......................................................................uagcacUauuugaaaucaguuc....................    1       1    Fm1
    ......................................................................uagcaccauuugaaauUaguuc....................    1       1    Fm1
    ......................................................................uagcaccauuugaaaucCguuc....................    1       1    Fm1
    ......................................................................uGgcaccauuugaaaucaguuc....................    3       1    Fm1
    ......................................................................uagcaccauuugaaaucaguuc....................    115     0    Fm1
    ......................................................................uagcaccauuugaaaucGguuc....................    1       1    Fm1
    ......................................................................uagcaccauuugaaaucaguGca...................    3       1    Fm1
    ......................................................................uagcaccauuugaaaucaguucU...................    1       1    Fm1

    ......................................................................uagcaccauuugaaaucagu......................    3       0    Mm1
    ......................................................................uagcaccauuugaaaucaguu.....................    81      0    Mm1
    ......................................................................uagcaccauuugaaauAaguu.....................    1       1    Mm1
    ......................................................................uagcaccauuugaaaucCguu.....................    1       1    Mm1
    ......................................................................uagcaccauuugaaaucGguuc....................    1       1    Mm1
```

Star Mature

```
ggagacuuuuuggccgugauugccugucaaguguacuguuuucauuuguugcauagaguuauugaccaucuagcaccauuugaaaucaguucauuggagaggacaugcugcg
......................................................................uagcaccauuugaaaucaguuc....................   138   0   Mm1
......................................................................uGgcaccauuugaaaucaguuc....................   1     1   Mm1
......................................................................uagcaccauuugaaaucaguuA....................   9     1   Mm1
......................................................................uagcaccauuugaaaucUguuc....................   1     1   Mm1
......................................................................uagcaccauuugaaaucaguuca...................   11    0   Mm1
......................................................................uagcaccauuugaaaucaguucU...................   3     1   Mm1
......................................................................uagcaccauuugaaaucaguGca...................   10    1   Mm1

..................................acuguuuucauuuguugcauaga.......................................................   7     0   Mm2
....................................uguuuucauuuguugcauaga.......................................................   1     0   Mm2
......................................................................uGgcaccauuugaaaucagu......................   3     1   Mm2
......................................................................uagcaccauuugaaaucagu......................   1     0   Mm2
......................................................................uagcaccauuugaaaucaguu.....................   9     0   Mm2
......................................................................Nagcaccauuugaaaucaguuc....................   1     1   Mm2
......................................................................uagcaccauuGgaaaucaguuc....................   1     1   Mm2
......................................................................uUgcaccauuugaaaucaguuc....................   1     1   Mm2
......................................................................uagcaccauuugaaauGaguuc....................   1     1   Mm2
......................................................................uagcaccauuugaaauAaguuc....................   4     1   Mm2
......................................................................uagcaccauuugaaaucaguAc....................   1     1   Mm2
......................................................................uagcaccauuugaaaucaguuU....................   1     1   Mm2
......................................................................uagcaccauuAgaaaucaguuc....................   1     1   Mm2
......................................................................uagcaccauuugaaaucaUuuc....................   1     1   Mm2
......................................................................uagcaccauuugaaaucGguuc....................   2     1   Mm2
......................................................................uagcaccauuugaaaucCguuc....................   1     1   Mm2
......................................................................uagcaccauuugGaaucaguuc....................   1     1   Mm2
......................................................................uagcaccauuugaaaucaguuc....................   361   0   Mm2
......................................................................uagcaccauuugaaaucaguGca...................   1     1   Mm2
......................................................................uagcaccauuugaaaucaguuca...................   5     0   Mm2
......................................................................uagcaccauuugaaaucaguucU...................   17    1   Mm2

..............cgugauugccugucaagugu..............................................................................   4     0   Fm3
..................................acuguuuucauuuguugcauaga.......................................................   28    0   Fm3
..................................acuguuuucauuuguugGauaga.......................................................   2     1   Fm3
...................................cuguuuucauuuguugcauaga.......................................................   2     0   Fm3
.....................................................................cuagcaccauuugaaaucaguuc....................   1     0   Fm3
......................................................................uagcaccauuugaaaucag.......................   52    0   Fm3
......................................................................uagcaccauuugaaaucaU.......................   1     1   Fm3
......................................................................uGgcaccauuugaaaucagu......................   1     1   Fm3
......................................................................uagcaccauuuAaaaucagu......................   1     1   Fm3
......................................................................uagcaccauuugaaaucagu......................   27    0   Fm3
......................................................................uaUcaccauuugaaaucagu......................   4     1   Fm3
......................................................................uagcaccauuugaaauAaguu.....................   1     1   Fm3
......................................................................uagcaccauuugaaaucaguA.....................   1     1   Fm3
......................................................................uagcaccauuugaaaucaguu.....................   92    0   Fm3
......................................................................uagcaccauuugUaaucaguu.....................   1     1   Fm3
......................................................................uagcaccauuugaaaucGguu.....................   1     1   Fm3
......................................................................uagcaccauuugaaauUaguuc....................   1     1   Fm3
......................................................................uaAcaccauuugaaaucaguuc....................   2     1   Fm3
......................................................................uagcaccauuugaaaucaguuc....................   438   0   Fm3
......................................................................uagcaccauuugaaaucaguuA....................   3     1   Fm3
......................................................................uagcaccauuugaaaucaguAc....................   1     1   Fm3
......................................................................uagcaccauuugaaauGaguuc....................   1     1   Fm3
......................................................................uagcaccauuGgaaaucaguuc....................   1     1   Fm3
......................................................................uagcaccauuugaaauAaguuc....................   2     1   Fm3
......................................................................uagcaccauuugaaaucCguuc....................   1     1   Fm3
......................................................................uagcaccauuugaaaucaguuca...................   4     0   Fm3
......................................................................uagcaccauuugaaaucaguucU...................   8     1   Fm3
.......................................................................agcaccauuugaaaucaguuc....................   1     0   Fm3

..................................acuguuuucauuuguugcauaga.......................................................   7     0   Mm3
..................................acuguuuucauuuguugGauaga.......................................................   1     1   Mm3
..............................................uguugcauagaguuauuCaccauc..........................................   1     1   Mm3
......................................................................uagcaccauuugaaaucagu......................   16    0   Mm3
......................................................................uGgcaccauuugaaaucagu......................   7     1   Mm3
......................................................................uagcaccauuugaaauAaguu.....................   1     1   Mm3
......................................................................uagcaccauuugaaaucaguu.....................   14    0   Mm3
......................................................................uagcaccUuuugaaaucaguuc....................   1     1   Mm3
......................................................................uagcaccauuGgaaaucaguuc....................   1     1   Mm3
......................................................................uagcaccauuugaUaucaguuc....................   1     1   Mm3
......................................................................uagcaccauuugaaaucCguuc....................   4     1   Mm3
```

```
                                 Star                              Mature
ggagacuuuuuggccgugauugccugucaaguguacuguuuucauuuguugcauagaguuauugaccaucuagcaccauuugaaaucaguucauuggagaggacaugcugcg
......................................................................uagcaccauuugaaaucaguuU....................    4     1     Mm3
......................................................................uagcaccauuugaaauAaguuc....................    4     1     Mm3
......................................................................uagcaccauuugaaaucaguuc....................    570   0     Mm3
......................................................................uagcaccauuugaaauGaguuc....................    1     1     Mm3
......................................................................Nagcaccauuugaaaucaguuc....................    1     1     Mm3
......................................................................uagcaccauuugaaaucaguuA....................    2     1     Mm3
......................................................................uagcaccauuugaaaucaguGca...................    2     1     Mm3
......................................................................uagcaccauuugaaaucaguucU...................    28    1     Mm3
......................................................................uagcaccauuugaaaucaguuca...................    8     0     Mm3
......................................................................uagcaccauuugaaaucaguucUuu.................    1     1     Mm3
```
